# A cause and protective treatment for acute and progressive disability and grey matter atrophy

**DOI:** 10.1093/brain/awaf465

**Published:** 2025-12-15

**Authors:** Erika A Aguzzi, Roshni A Desai, Zhiyuan Yang, Andrew L Davies, Don Mahad, Bernard Siow, Ayse G Yenicelik, Radha Desai, Eleni Giama, AlBeshr Almasri, Miranda Colman, Celine Geywitz, Lucas Schirmer, Paul A Felts, Kenneth J Smith

**Affiliations:** Department of Neuroinflammation, UCL Queen Square Institute of Neurology, London WC1N 3BG, UK; Department of Neuroinflammation, UCL Queen Square Institute of Neurology, London WC1N 3BG, UK; Department of Neuroinflammation, UCL Queen Square Institute of Neurology, London WC1N 3BG, UK; Department of Neuroinflammation, UCL Queen Square Institute of Neurology, London WC1N 3BG, UK; Centre for Clinical Brain Sciences, University of Edinburgh, Edinburgh EH16 4SB, UK; In Vivo Imaging, Biological Research Facility, The Francis Crick Institute, London NW1 1AT, UK; Department of Neuroinflammation, UCL Queen Square Institute of Neurology, London WC1N 3BG, UK; Department of Neuroinflammation, UCL Queen Square Institute of Neurology, London WC1N 3BG, UK; Department of Neuroinflammation, UCL Queen Square Institute of Neurology, London WC1N 3BG, UK; Department of Neuroinflammation, UCL Queen Square Institute of Neurology, London WC1N 3BG, UK; Department of Neuroinflammation, UCL Queen Square Institute of Neurology, London WC1N 3BG, UK; Division of Neuroimmunology, Department of Neurology, Medical Faculty Mannheim, Heidelberg University, Mannheim 68167, Germany; Division of Neuroimmunology, Department of Neurology, Medical Faculty Mannheim, Heidelberg University, Mannheim 68167, Germany; Mannheim Center for Translational Neuroscience, Heidelberg University, Mannheim 68167, Germany; Interdisciplinary Center for Neurosciences, Heidelberg University, Heidelberg 69120, Germany; Centre for Anatomy and Human Identification, University of Dundee, Dundee DD1 4HN, UK; Department of Neuroinflammation, UCL Queen Square Institute of Neurology, London WC1N 3BG, UK

**Keywords:** neuroinflammation, neurodegeneration, grey matter atrophy, neuroprotection, disease progression, relapse

## Abstract

Acutely inflamed CNS lesions can be sufficiently hypoxic to cause temporary neurological disability. A new experimental lesion reveals that brief hypoxia can also ignite a slow-burning atrophy of the grey matter, resulting in a lifetime of slowly progressive disability. The progressive disability eventually exceeds that observed acutely, indicating that acutely functioning tissue can nevertheless be destined to atrophy.

Remarkably, both the temporary initial disability and the ensuing progressive disability and atrophy are significantly reduced if the acute hypoxia is avoided by four days of treatment with vasodilating nimodipine, or by simply breathing raised oxygen concentration.

Thus, a lifetime of progressive disability and neurodegeneration can be the legacy of a few days of inflammatory hypoxia experienced in young adulthood, and avoided by maintaining lesion oxygenation. The findings may help to understand and treat some progressive neurological disorders, including multiple sclerosis.

## Teaser

Aguzzi *et al.* show in an experimental lesion model that a few days of inflammatory hypoxia in young adulthood can prime grey matter for subsequent slowly progressive atrophy. Preventing this early hypoxia—through improved oxygenation or vasodilatory treatment—reduced both the initial deficits and the later progressive decline.

## Introduction

Slowly progressive (‘smouldering’) neurodegeneration and atrophy characterize several neurological disorders, including progressive multiple sclerosis and Alzheimer’s disease,^1^ but the mechanisms responsible are not understood. A key outstanding question is whether the progressive degeneration in later life is due to contemporaneous events in later life, or whether it can be the delayed expression of damage incurred much earlier. For example, various features of innate neuroinflammation (whether or not provoked by autoimmunity) have been implicated in chronic ongoing neurodegeneration in multiple sclerosis. These include microglial activation, blood–brain barrier (BBB) breakdown, actual and virtual tissue hypoxia, mitochondrial impairment especially of respiratory complex IV, and the production of nitric oxide and reactive oxygen species (ROS).^2-6^ However, it remains uncertain whether brief acute exposure to these same factors earlier in life may also result in neurodegeneration but after a substantial delay. It is correspondingly unclear whether acutely targeting any of these factors in early life may provide a route to achieve protection from later progressive neurodegeneration and disability.

Research has been hindered by a paucity of experimental animal models in which to study the mechanisms and test potential therapies. Here we introduce a model of slowly progressive disability, neurodegeneration and atrophy, and use it to identify a strategy to achieve significant lifetime protection from disability and pathology. The lesion is induced in the grey matter of the spinal cord because many studies have shown that, in multiple sclerosis at least, it is atrophy of the grey matter that is pivotal in determining disability,^7^ and such atrophy in the spinal cord, rather than brain, has greater association with advancing disability.^8^

To explore mechanisms potentially responsible for the slowly progressive disability, and whether targeting the mechanisms may provide a neuroprotective treatment, we considered particular features of the acute lesion. The acutely inflamed lesion labels strongly for markers of hypoxia, which have been shown in other models^9-12^ to cause acute and reversible neurological deficits, and to be reversed by treatment with the vasodilating agent nimodipine or by breathing raised oxygen.^9,11,12^ We have therefore examined whether these agents may not only reduce the acute disability in the new lesion, but also protect from the progressive accumulation of permanent disability.

## Materials and methods

The methods are described in detail in the Supplementary material.

### Lesion induction and assessment of neurological deficit

Young adult male Sprague Dawley rats were anaesthetized (isoflurane; 1.5%–2% in room air) and an intraspinal microinjection of the proinflammatory agent lipopolysaccharide (LPS; 0.5 μl of 80 ng/µl in saline) performed into the right ventral horn at the T13 vertebra using methods similar to those previously described.^13^ Control animals received similar injections of saline alone. The wound was closed and anaesthesia discontinued. Neurological function was assessed by analysis of gait and tail elevation using video recordings of animals walking freely on a level surface both before lesion induction, and at various times afterwards until termination 6 or 12 months later.

### Treatment

Treatments were selected based on observations from the acutely inflamed lesion, which was profoundly hypoxic. Based on our previous observations in other neuroinflammatory and hypoxic lesions,^9-12^ animals were randomized into groups prior to lesion induction, and treated for just the first 4 days with either the CNS-selective vasodilating agent nimodipine or inspiratory normobaric oxygen (80%). Both these treatments promptly improve the oxygenation of inflamed CNS tissue.^9^

For nimodipine, animals received an intraperitoneal injection while under the surgical anaesthesia (30 mg/kg) and then seven additional doses (30 mg/kg) by twice daily oral gavage. Animals treated with oxygen (50% or 80% as noted) were placed after anaesthesia in an environment of raised oxygen (50% or 80% as noted) for 4 days. Controls received either vehicle or room air, as appropriate. All treatment was discontinued after 4 days and animals were returned to their home cages and maintained at room air with food and water *ad libitum*.

### Tissue collection and histological examination

At termination, all animals were deeply anaesthetized (3% isoflurane) and transcardially perfused with paraformaldehyde (4% in 0.15 M phosphate buffer). In some animals, pimonidazole (60 mg/kg; intravenously) was administered 4 h prior to perfusion to assess tissue oxygenation. The spinal cord at the site of the injection was removed and post-fixed in 4% paraformaldehyde overnight, prior to cryoprotection and snap freezing in liquid nitrogen/isopentane. For histochemical examination of mitochondrial complex activity, some animals were transcardially perfused with cold, oxygenated rinse solution alone, and the fresh tissue was removed and snap frozen as described earlier. Lesions were collected from animals in matched pairs (treatment and control).

For (immuno)histochemical examination, fixed cryosections were examined at the lesion epicentre using standard techniques and a range of stains and antibodies (Supplementary Tables 1 and 2). Images were obtained using standard light and confocal microscopes. All analysis and quantification was performed blind using ImageJ.

In some experiments, lesion volumes were assessed by studying excised spinal cords positioned in a 9.4 T preclinical MRI scanner.

### Mitochondrial studies

Mitochondrial biogenesis was assessed following the intraperitoneal administration of bromodeoxyuridine (200 mg/ml; 5 ml/kg). Control animals were injected with saline. At termination, tissues were fixed by perfusion as described above and mitochondrial biogenesis revealed by immunohistochemistry.

Histochemical study of mitochondrial complex IV was conducted as previously described.^14^

## Results

**Figure 1:**
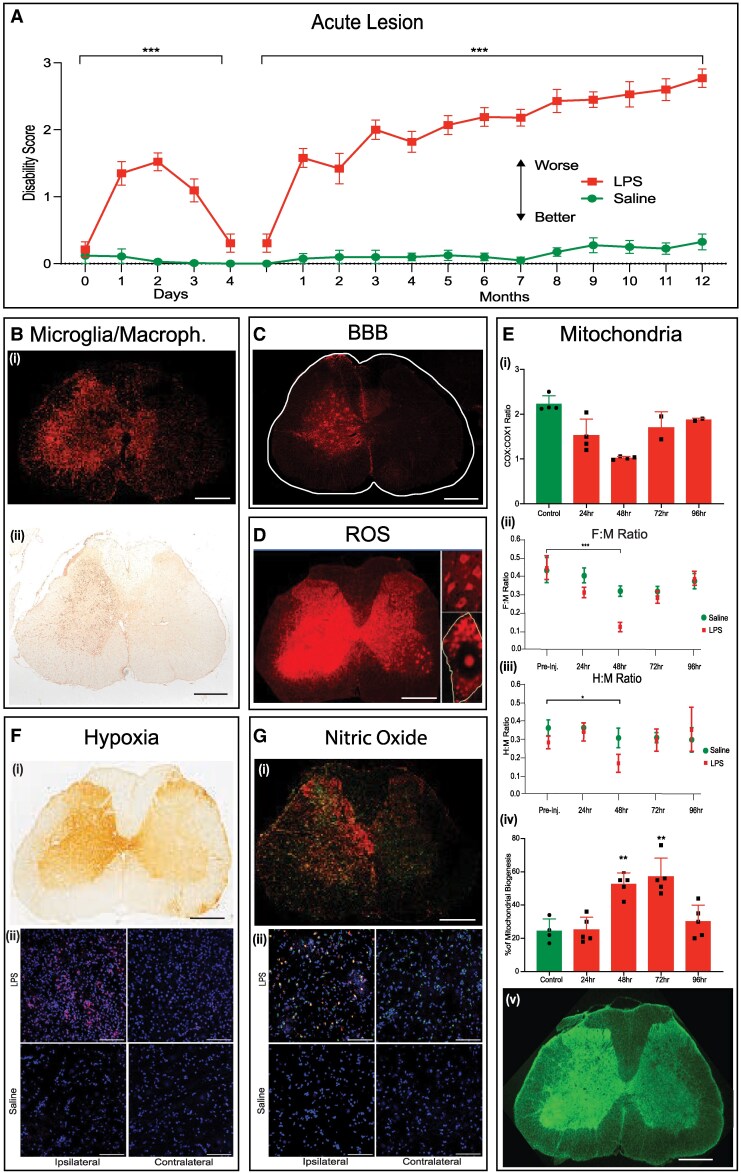
**Progression of the lesion: disability and acute histological and electrophysiological findings.** (**A**) Course of disability observed while rats are walking on a horizontal surface for 1 m, showing a significant difference between animals injected with lipolysaccharide (LPS; *n* = 20) versus saline (*n* = 10) (*P* < 0.001). Rats normally walk with raised tail (score 0), but weakness causes the distal tail to droop and touch the ground (score 1.5) or the whole tail can become flaccid (score 3). Mean ± standard error of the mean, *P* < 0.001. Two-way ANOVA with Bonferroni correction for multiple comparisons. [**B**–**G** and **E**(**v**)] Histological images showing transverse sections through the spinal cord at the level of an intraspinal injection made into the left side as shown. [**B**(**i** and **ii**)] The lesioned tissue is characterized by raised microglial/macrophage activation on the lesioned side, marked by immunolabelling for IBA1 [**B**(**i**)] and ED1 [**B**(**ii**)] 2 days post-injection (dpi) of LPS. [**F**(**i** and **ii**)] The lesion is acutely hypoxic, indicated by immunolabelling for pimonidazole [**F**(**i**)] and hypoxia inducible factor-1a (HIF1a) [**F**(**ii**)] at 3 dpi. (**C**) There is breakdown of the blood–brain barrier largely confined to the lesioned side, marked by immunolabelling for fibrinogen 3 dpi. (**D**) The lesion shows raised production of superoxide, indicated by dihydroethidium fluorescence at 3 dpi, which is especially prominent in ventral horn motor neurons (*top inset*) and in clusters of mitochondria within such neurons [*bottom inset* shows a single motor neuron (outlined)]. [**G**(**i** and **ii**)]. The lesioned side shows marked expression of cells immunolabelled for inducible nitric oxide synthase (iNOS; red) and ED1 (green) at 1 dpi, indicating raised production of nitric oxide especially in activated microglia/macrophages (appearing yellow). iNOS^+^ cells are rare on the contralateral side in LPS-lesioned tissue, and absent from control spinal cords injected with saline [**G**(**ii**)]. The free radicals superoxide and nitric oxide will avidly combine to form peroxynitrite, and all these three factors impair mitochondrial function, which will be accentuated by the hypoxia. [**E**(**i**)] Bar graph showing the corresponding significant impairment of mitochondrial function in motor neurons during the first 4 days, indicated by changes in the ratio of histochemical reactivity of the mitochondrial complex IV (COX) and the immunoreactivity of the catalytic COX complex I. Data-points indicate individual animals. Mean ± standard deviation, one-way ANOVA. [**E**(**ii** and **iii**)] The depression of mitochondrial function correlates with a depression of the excitability of motor neurons, manifest as a reduction in the ratio of the electromyographic F wave compared with the directly conducted M response over time [**E**(**ii**)], and a reduction in the ratio of the electromyographic H reflex compared with the directly conducted M response over time [**E**(**iii**)], both of which provide a measure of the excitability of ventral horn motor neurons. Excitability is significantly depressed {*P* < 0.001 [**E**(**ii**)] and *P* < 0.05 [**E**(**iii**)]} at 2 dpi, when mitochondrial function is also significantly depressed [**E**(**i**)]. [**E**(**iv**)] The depression of mitochondrial function on Days 2–3 is accompanied by a significant (*P* < 0.01) increase in mitochondrial proliferation on Days 2–3 indicated by increased bromodeoxyuridine (BrDU) labelling of mitochondrial DNA. Mean ± standard deviation; one-way ANOVA, with Dunnett's correction for multiple comparisons. [**E**(**v**)] The marked increase in mitochondrial proliferation is associated with a marked increase in mitochondrial density on the lesioned side, indicated by immunolabelling for mitochondrial porin at 3 dpi). *P < 0.05; **P < 0.01; ***P < 0.001; ****P < 0.0001. Scale bars = 500 μm in **B**(**i** and **ii**), **C**, **D**, **E**(**v**), **F**(**i**), **G**(**i**); 100 μm in **F**(**ii**) and **G**(**ii**).

**Figure 2:**
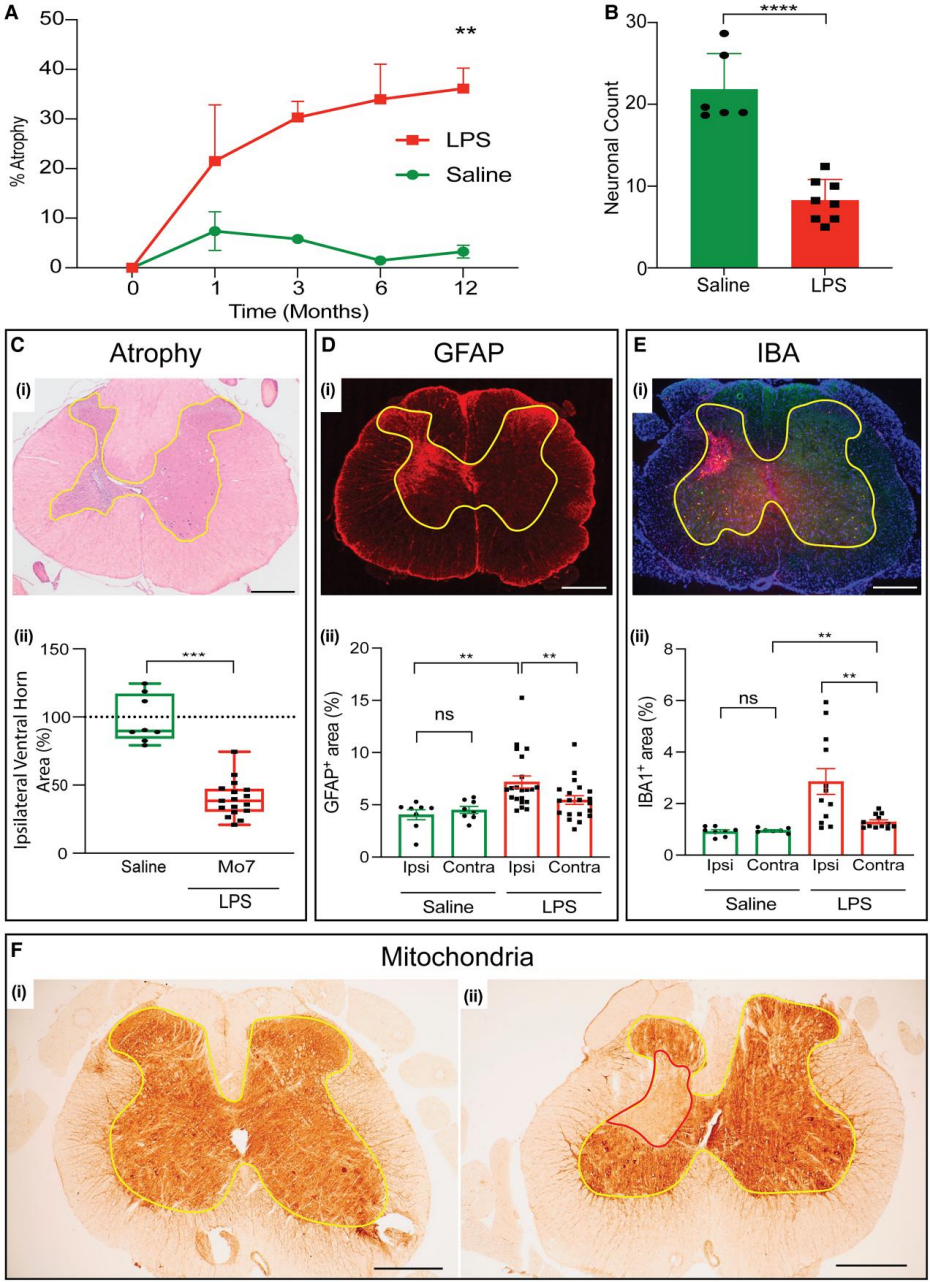
**Progression of the lesion: chronic findings**. (**A**) Graph showing the increasing atrophy of the grey matter at the lesion site, comparing ipsilateral versus contralateral sides, and injections of lipopolysaccharide (LPS) versus saline. LPS: 1 m (*n* = 3), 3 m (*n* = 3), 6 m (*n* = 4) and 12 m (*n* = 12). *T*-test, *P* = 0.006. (**B**) Graph demonstrating significant loss of motor neurons at 12 months, comparing animals injected with LPS (*n* = 8) versus saline (*n* = 6). Mean ± standard deviation, *t*-test, *P* = 0.0001. [**C**(**i**) and **D**(**i**)] Illustrations showing atrophy of the ipsilateral ventral horn. [**C**(**i**)] Transverse haematoxylin and eosin section through an LPS lesion at 12 months illustrating marked atrophy of the ipsilateral grey matter (grey matter boundary outlined in yellow). [**C**(**ii**)] Graph showing significant atrophy of the ipsilateral ventral horn at 7 months in animals injected with LPS versus saline. Independent *t*-test (*P* < 0.001). [**D**(**i** and **ii**)] Illustrations showing astrogliosis at the site of injection of LPS. [**D**(**i**)] Transverse section from a spinal cord injected with LPS 12 months previously, showing immunolabelling for glial fibrillary acidic protein (GFAP). [**D**(**ii**)] Graph showing significantly increased immunolabelling for GFAP ipsilateral to the site of injection, and compared with saline-injected controls. Independent *t*-test (*P* < 0.01). [**E**(**i and ii**)] Illustrations showing concentration and activation of microglia/macrophages in chronic lesions at the site of LPS injection. [**E**(**i**)] Transverse section from spinal cord injected with LPS 12 months previously, showing immunolabelling for IBA1, a marker of microglia/macrophages. [**E**(**ii**)] Graph showing significantly increased immunolabelling for IBA1 ipsilateral to the site of injection, and compared with saline-injected controls. LPS versus ipsilateral saline, independent *t*-test (*P* < 0.01); LPS ipsilateral versus contralateral, paired *t*-test (*P* < 0.01). [**F**(**i and ii**)] Transverse sections from saline- and LPS-injected spinal cords showing histochemistry for mitochondrial complex IV activity, 12 months post-injection. The grey matter boundary is indicated in yellow. Note the lack of complex IV activity in the grey matter undergoing atrophy (outlined in red). The injections in **C**(**i**), **D**(**i**), **E**(**i**) and **F** were positioned more superficially in the spinal cord than in most other figures. Scale bars = 500 μm in **C**(**i**), **D**(**i**), **E**(**i**) and **F**(**i** and **ii**).

The lesion and associated disability were found to evolve over three phases (Fig. 1A). First, an acute inflammatory lesion (Days 1–4) accompanied by significant (*P* < 0.001) relapse-like hindlimb and tail weakness. Second, a period of remission as the inflammation subsided and function was restored. Finally, the onset of progressive significant disability (*P* < 0.001) that evolved over the lifetime in step with progressive atrophy of the previously affected grey matter (Fig. 2A). The eventual disability exceeded that observed acutely. Saline-injected control animals showed no such changes.

The progressive atrophy commenced within a month of the initial inflammatory lesion, which focused attention on events in the acute lesion that may be responsible. Histological examination of the acute lesion revealed prominent activation and recruitment of microglia/macrophages in the grey matter on the lesioned side of the spinal cord [Fig. 1B(i and ii) and Supplementary Fig. 1B], which was most intense on Days 2–3 post-injection, coinciding with the acute peak in disability. At this time the inflamed tissue was noticeably hypoxic, as indicated by significant immuno-labelling for pimonidazole (*P* ≥ 0.001) [Fig. 1F(i) and Supplementary Fig. 1C] and hypoxia inducible factor-1α (HIF-1α; *P* < 0.05) [Fig. 1F(ii) and Supplementary Fig. 1D]. The BBB on the side ipsilateral to the lesion was acutely significantly compromised (Fig. 1C and Supplementary Fig. 1F) (*P* < 0.001, compared with the contralateral side; *P* < 0.05, compared with saline-injected controls). The hypoxic tissue showed increased labelling for superoxide/ROS (Fig. 1D), apparently within mitochondria (Fig. 1D, inset). Notably, the rise in superoxide/ROS was coupled at 2–3 days with the significant unilateral expression of the inducible form of nitric oxide synthase, and thus of nitric oxide [Fig. 1G(i and ii) and Supplementary Fig. 1E] (*P* < 0.01, compared with the contralateral side; *P* < 0.05, compared with saline-injected controls), and the inevitable formation (with superoxide) of peroxynitrite.^15^ Superoxide, nitric oxide and peroxynitrite all impair mitochondrial function,^16^ and this was evidenced in motor neurons by a significant decrease of 63% on Day 2 of the ratio of the histochemical activity of mitochondrial complex IV (the oxygen-binding complex) and the immunohistological labelling for the catalytic COX1 subunit of complex IV [Fig. 1E(i) and Supplementary Fig. 1G] (*P* < 0.01), denoting impaired mitochondrial function. The maximal mitochondrial impairment on Day 2 precisely coincided with the peak of acute disability (Fig. 1A) and impaired electrical excitability of motor neurons [Fig. 1E(ii and iii)]. The fall in mitochondrial function and presumed tissue energy crisis was promptly (Days 2 and 3) followed by a marked (120%) increase in mitochondrial biogenesis^17^ [Fig. 1E(iv)] (*P* < 0.01) and a clear unilateral increase in abundance of mitochondria [Fig. 1E(v)]. The acute inflammation and hypoxia substantially subsided by Day 4, coincident with the onset of remission and the return of near normal function, which persisted for approximately 2 weeks.

**Figure 3:**
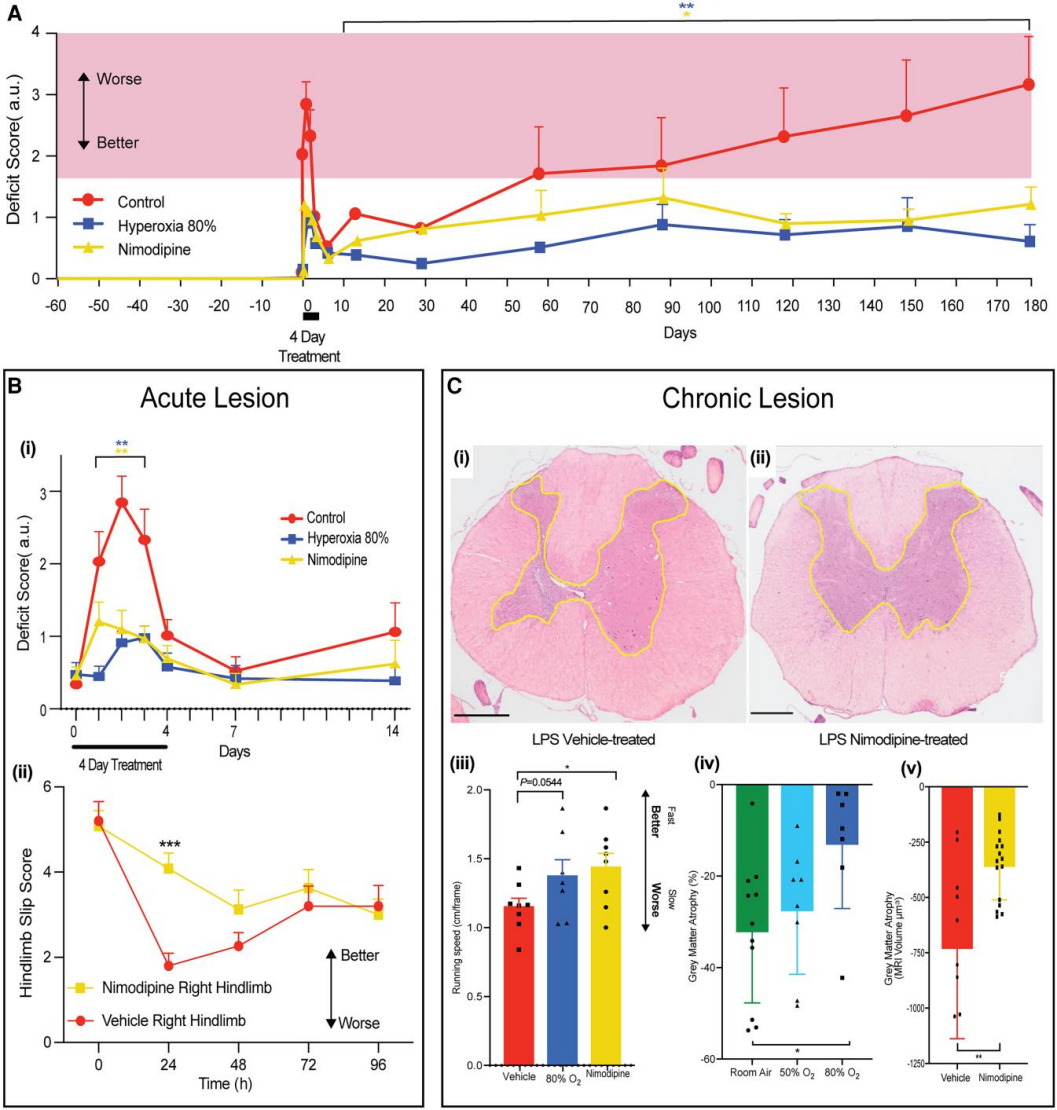
**Protection by treatments that prevent lesion hypoxia.** (**A**) Course of disability comparing animals injected with lipopolysaccharide (LPS) and treated for only the first 4 days with either nimodipine (*n* = 23), 80% inspiratory oxygen (*n* = 23), or no treatment (*n* = 23). The pink band marks the severity of disability that is easily detected by visual observation while rats are walking on a level surface. Note that untreated animals spend much of their time in the pink region, but treated animals are never this severely affected. Mean ± standard error of the mean (SEM); one-way ANOVA, Dunnett's multiple comparisons test; hyperoxia, *P* = 0.0045, nimodipine *P* = 0.0362. [**B**(**i**)] Detail from Fig. 3A. Mean ± SEM; two-way ANOVA, Dunnett's multiple comparisons test; hyperoxia, *P* < 0.01; nimodipine *P* < 0.01. [**B**(**ii**)] Graph showing hindlimb disability marked by slips of the right foot while walking along a horizontal ladder, for animals injected with LPS into the right ventral horn and either treated with nimodipine (*n* = 24) or vehicle (control; *n* = 15). Animals treated with nimodipine walked significantly better than untreated animals 2 days after LPS injection. Animals were trained from 1 day before surgery (baseline) up to 4 days after surgery. Two-way ANOVA, *P* < 0.001. Values are means ± SEM. [**C**(**i** and **ii**)] Transverse sections through the spinal cord at the level of lesions induced 12 months previously, comparing vehicle control treatment [same as Fig. 2C(i)] with protective treatment for the first 4 days alone with nimodipine. The border of the grey matter is outlined in yellow. Nimodipine treatment protects from grey matter atrophy. Scale bars = 500 μm. [**C**(**iii**)] Graph showing the walking speeds of rats freely walking on a flat surface recorded by video camera 6 months after LPS injection, comparing untreated control animals with those acutely treated (for 4 days) with inspiratory 80% oxygen, or nimodipine. Rats treated with nimodipine walked significantly faster than untreated animals. Mean ± SEM; independent *t*-test; *P* < 0.05. [**C**(**iv** and **v**)] Bar graphs showing significant protection from grey matter atrophy at 12 months following LPS injection, mediated by acute (4 days) treatment with [**C**(**iv**)] 50% or 80% inspiratory oxygen (room air controls *n* = 11; 50% oxygen *n* = 8; 80% oxygen *n* = 7) (atrophy determined from haematoxylin and eosin sections comparing lesioned tissue with vehicle controls; *P* < 0.05) or [**C**(**v**)] acute (4 days) treatment with nimodipine (*n* = 16) versus vehicle controls (*n* = 8) (grey matter volume determined by MRI; mean ± SD; one-way ANOVA; mean ± SD, *t*-test; *P* < 0.01).

The period of remission was followed by the third phase of lesion development characterized by the degeneration of motor neurons (Fig. 2B) (*P* < 0.0001) and the slowly progressive atrophy of the previously inflamed spinal grey matter [Fig. 2A and C(i and ii)] (*P* < 0.001). The degeneration occurred in association with significant focal accumulation of astrocytes [Fig. 2D(i and ii)] (*P* < 0.01) and significant activation of (iNOS^−^) microglia/macrophages [Fig. 2E(i and ii)] (*P* < 0.01), and, notably, the tissue undergoing atrophy showed mitochondrial damage manifest as a deficiency of the activity of mitochondrial respiratory complex IV [Fig. 2F(ii)]. The advancing atrophy occurred hand in hand with the gradual return of significant disability over the lifetime, manifest as a limp and other gait abnormalities in the hindlimb ipsilateral to the lesion [Fig. 3C(iii) and Supplementary Fig. 1J(ii)] (*P* < 0.05), coupled with weakness of the tail (Fig. 1A) (*P* < 0.001).

Animals treated with nimodipine or oxygen for the first 4 days following lesion induction showed a significant reduction in the first, acute phase of disability [Fig. 3A and B(i and ii) and Supplementary Fig. 1A; oxygen *P* < 0.01; nimodipine *P* < 0.01 and *P* < 0.001]. Although reducing disability, there was no significant difference in the magnitude of acute inflammation, as judged by the abundance of microglia/macrophages (Supplementary Fig. 1H), or their amoeboid, activated morphology (not shown). Even though the treatments were applied for just the first 4 days following lesion induction, treated animals showed a significant protection from not only the acute disability, but also significant protection from the subsequent lifetime of progressive disability [Fig. 3A; oxygen *P* < 0.01; nimodipine *P* < 0.05; Fig. 3C(iii) and Supplementary Fig. 1A; nimodipine *P* < 0.0001; oxygen *P* < 0.01], and grey matter atrophy [Fig. 3C(i, ii, iv and v) and Supplementary Fig. 1J(i)] (oxygen *P* < 0.05 and *P* < 0.01, nimodipine *P* < 0.01 and *P* < 0.05). In untreated animals the acute and chronic disability was sufficiently severe to be apparent upon simple visual inspection of freely walking animals, but in animals treated with nimodipine or oxygen any disability was subtle, with no significant difference in, for example, balance (Supplementary Fig. 1I) or swing speed [Supplementary Fig. 1J(ii)], between the legs ipsilateral and contralateral to the lesion. Animals treated with nimodipine naturally walked significantly faster than untreated controls at 6 months (*P* < 0.05) (oxygen *P* = 0.054) [Fig. 3C(iii)].

## Discussion

The results describe an acute neuroinflammatory lesion in young adulthood that not only causes acute disability comparable to a relapse in multiple sclerosis, followed by remission, but is followed by a lifetime of slowly progressive disability. The progressive disability occurs in parallel with advancing neurodegeneration and atrophy of the previously inflamed tissue. The tissue undergoing progressive atrophy is characterized by astrocytic and microglial/macrophage activation and a loss of activity of mitochondrial complex IV, as occurs in multiple sclerosis.^18^ The acutely inflamed lesion is profoundly hypoxic, and treatment with nimodipine or oxygen (known to prevent hypoxia in another neuroinflammatory lesion^9^), not only significantly reduces the acute disability, but, importantly, it also significantly reduces the chronic, progressive disability. In fact, whereas in untreated animals the acute and chronic disability was sufficiently severe to be apparent upon simple visual inspection of freely walking animals (a level of disability that may prompt use of a walking stick in humans), in animals treated with nimodipine or oxygen any disability was subtle with no significant difference in, for example, swing speed, between the legs ipsilateral and contralateral to the lesion.

Considering mechanisms, the hypoxia of the acutely inflamed tissue appears key to several features of the lesion. The hypoxia will, for example, contribute to mitochondrial failure and thus to the acute disability, as it does in experimental autoimmune encephalomyelitis (EAE, a common model of multiple sclerosis).^9,11,19,20^ Indeed, the hypoxia appears to be a main cause of the acute disability given that the disability is significantly reduced by treatment with vasodilating nimodipine or oxygen. The hypoxia will also promote other features of the lesion, including the formation of the observed microglial/macrophage activation^21^ and BBB breakdown,^22^ and the production of ROS^23^ and nitric oxide,^23^ and hence the production of the peroxynitrite^15^ that can damage mitochondrial complexes and DNA.^24^ Thus the hypoxia lies as a root cause of several factors that synergize to create a particularly toxic cellular environment,^25^ and one that is particularly deleterious for oxidative mitochondrial metabolism.^26^ The consequent shortage of ATP will depolarize neurons, significantly reducing their excitability as observed, and resulting in the observed neurological deficits. In common with EAE,^9,11,12^ it appears that the hypoxia is not sufficiently severe to kill the neurons and other cells, and when the inflammation subsides and oxygenation increases the cells resume functioning and remission commences.

The chronic, slowly progressive accumulation of permanent disability can be attributed to the slowly progressive neurodegeneration and the grey matter atrophy that accompanies it, but why the cells degenerate is uncertain. The fact that the degeneration is significantly reduced by treatment with nimodipine or oxygen restricted to the first 4 days of lesion development appears to pinpoint the seeds of the damage being sown within the acutely inflamed hypoxic lesion during young adulthood. Nimodipine has a range of potentially valuable neuroprotective properties that may be involved,^27,28^ but the fact that oxygen is similarly protective focuses attention on the vasodilatory effects of nimodipine which, like inspiratory oxygen, will improve tissue oxygenation and overcome the hypoxia.

If the damage is inflicted in the acute hypoxic lesion in early adulthood it implies the existence of a cellular mechanism capable of storing the ‘memory’ of the earlier event over time, while the cells continue functioning. A mechanism that fits the available evidence derives from the cascade described above in which hypoxia promotes a toxic environment including the excessive production of mitochondrial superoxide, nitric oxide and peroxynitrite. Peroxynitrite, in particular, can damage mitochondrial complex IV^26^ and the mtDNA^24^ responsible for the production of functioning complex IV (and other essential mitochondrial constituents), consistent with the loss of mitochondrial complex IV activity observed in the tissue undergoing atrophy. Indeed, the toxic environment for mitochondria occurs at precisely the time of the dramatic increase in mitochondrial biogenesis and mtDNA replication on Days 2 and 3 following LPS injection, and thus the newly forming mtDNA is likely to be damaged. Replication of the damaged mtDNA over time as mitochondria proliferate will increase heteroplasmy, eventually reaching a lethal point where the cells lack sufficient capacity for oxidative phosphorylation. Thus, it appears that the cellular ‘memory’ of the acute damage in young adulthood may be stored as permanent damage to the cellular mtDNA.

It is interesting that the eventual disability exceeds that observed acutely, indicating that the tissue undergoing neurodegeneration and atrophy includes tissue that continued functioning throughout the period of acute inflammation. This observation suggests that the mechanisms responsible for the degeneration can be established in the absence of preceding deficits, raising the possibility that tissue can remain functioning and asymptomatic despite having been mortally damaged. The mechanisms involved may help to explain the ‘progression independent of relapsing activity’ (PIRA) described in progressive multiple sclerosis^29^ and other neurodegenerative diseases that arise in later life without preceding neurological deficits.

In summary, the findings show that inflamed hypoxic lesions in young adulthood not only cause acute neurological deficits that diminish as the inflammation and hypoxia subside, but the hypoxic inflamed lesions also covertly ‘doom’ the affected tissue to eventual degeneration over the lifetime, in association with a loss of activity of mitochondrial complex IV. The acute and chronic disability, and progressive degeneration and atrophy are all significantly reduced by either of two strategies that protect the inflamed tissue from the associated acute hypoxia. It is intriguing that just a few days of inflammatory hypoxia in young adulthood can inflict occult damage that resonates over the lifetime. Additionally, that treatment that protects from hypoxia for just these few days provides significant lifelong protection. It is encouraging that avoiding inflammatory hypoxia provides a relatively simple route to a treatment achieving both acute and chronic neuroprotection.

## Supplementary Material

awaf465_Supplementary_Data

## Data Availability

The data are available upon reasonable request to the corresponding author.
