# Supplementary material for: A cause and protective treatment for acute and progressive disability and grey matter atrophy
Source: Brain. 2025 Dec 15;149(4):1144–52. doi: 10.1093/brain/awaf465 (PMC13058463; doi:10.1093/brain/awaf465)
Supplement: awaf465_Supplementary_Data [file awaf465_supplementary_data.zip › brain-2025-00625-File006.pdf]

Online Supplementary text

Methods

Lesion Induction – Intraspinal LPS injection

A quarter laminectomy was performed aseptically between the T13 and L1 vertebrae in adult male Sprague Dawley rats (323.3+-48.7g, mean ± standard deviation), under deep isoflurane (Merail, Harlow, UK) anaesthesia (1.5-2% in room air). A glass micropipette was used to microinject lipopolysaccharide (LPS; 0.5 µl of 80 ng/µl in saline; *Salmonella enterica*, serotype typhimurium, Sigma-Aldrich, St Louis, MO) into the right ventral horn, positioned 700 µm lateral from the midline, at depths of 1100 and 1300 µm. Control animals received similar injections of saline alone. The injection site was marked with powdered charcoal on the dura for subsequent histological localisation.

We have chosen LPS as a pro-inflammatory agent because even in healthy humans, microglia are daily 'irritated and aggravated' by leakage into the brain of low levels of naturally circulating LPS, which contribute to a chronic state of low-grade neuroinflammation[1,2]. LPS is therefore a factor to be considered in the context of MS, especially where the blood-brain barrier is disturbed.

Assessment of neurological deficit

Video recordings were made of animals walking freely and voluntarily on a level surface to evaluate the function of the tail and hindlimbs at the following timepoints; before lesion induction, and at 1, 2, 3, 4, 7, and 14 days, and then monthly until termination at 6 or 12 months after lesion induction. Neurological function was assessed by gait analysis and by measuring tail elevation.

Gait Analysis

Gait analysis was assessed using established methods[3]. For each animal at each timepoint, a video recording was considered valid if the animal walked with steady speed for at least four consecutive steps. From this walk, stride length was measured, and stance and swing times were measured for both left and right hindlimbs. The percent stance time (i.e. duty factor) was calculated as stance time divided by stride time (duty factor = stance time / stride time), and balance between left and right hindlimbs as the duty factor of right hindlimb minus the duty factor of left hindlimb (balance = duty factor right - duty factor left). Additionally, swing speed was calculated as stride length divided by swing time (swing speed = stride length / swing time), stride speed as stride length divided by stride time (stride speed = stride length/ stride time), and running speed as an average of the stride speed of both hindlimbs.

Tail Elevation

The magnitude of voluntary tail elevation while walking was quantified using a customised scoring system modified from MacKenzie et al[4]. In brief, an overall score of tail elevation was awarded by observation of the video recorded for each animal at each timepoint, then a representative frame of the video was captured and used to measure the elevation of the tail tip and mid tail from tail base to adjust the overall score awarded from the video, if necessary. A higher score represents less tail elevation, indicating worse motor function.

Illustrations in Figures 3Bii and 3Ciii, and in Suppl. Figures I and Jii describe deficits in hindleg function, and Figures 1A and 3A, and Suppl. Figure A show deficits in tail function.

Treatment

Animals were randomised into groups prior to lesion induction, and treated for the first four days with nimodipine, or inspiratory normobaric oxygen (50% or 80% as noted), or vehicle or room air. For treatment with nimodipine, lesions were induced as described above and immediately following surgery the animals received an intraperitoneal (i.p.) injection of nimodipine (30mg/kg) or vehicle (ethanol:PEG400), and allowed to recover. Treatment with nimodipine (30mg/kg/dose) or vehicle was then maintained by twice daily oral gavage. Animals treated with normobaric oxygen or room air were housed in a chamber containing 50% or 80% (as noted) oxygen for the duration of treatment. All treatment was discontinued after four days, and all animals were returned to their home cages and maintained at room air with food and water *ad libitum* until perfusion in matched pairs (treatment and control).

Perfusion, Tissue Harvesting and Processing for Main (Immuno)-Histological Studies

Pimonidazole (60 mg/kg in saline; HPI Inc, Burlington, MA) was administered systemically via the saphenous vein under temporary light anaesthesia (1.5% isoflurane in room air) four hours prior to terminal perfusion, when required to assess tissue oxygenation. At termination, all animals were deeply anaesthetised (3% isoflurane) and transcardially perfused with rinse solution (0.9% NaCl, 2,000U/l heparin, 0.025% lidocaine, 0.02% 4-[2-hydroxyethyl]-1-piperazineethanesulfonic acid [pH 7.4]) followed by paraformaldehyde (4% in 0.15M phosphate buffer). Spinal cord tissue at the site of the injection was harvested and post-fixed in 4% paraformaldehyde overnight, prior to cryoprotection in 30% sucrose for 48 hours. To allow histochemical examination of the lesion, a subset of animals were transcardially perfused with cold, oxygenated rinse solution alone, and the fresh tissue was snap frozen in liquid nitrogen/isopentane.

Histology and Microscopy

For (immuno)histochemical examination, fixed, 12 µm-thick cryosections were examined at the lesion epicentre using standard techniques and a range of stains and antibodies (Supplementary Tables 1 & 2), as previously described[5]. Tissue labelled using the peroxidase detection system was viewed using an Axiophot light microscope (Carl Zeiss, Oberkochen, Germany) and photographed with a Nikon D300 camera (Nikon Instruments, Melville, NY). Fluorescent images were obtained using a Zeiss LSM5 Pascal or LSM710 confocal microscope, using x10, x20, x40 and x63 objectives.

Histological Quantification

All analysis and quantification was performed blind using ImageJ (NIH, Bethesda, MD). Quantification of ED1, IBA-1, GFAP, fibrinogen, iNOS and other labels was performed by counting the number of pixels above a set threshold, and expressed as the percentage of cross-sectional area coverage.

Pimonidazole labelling was quantified as described previously[5]. Analysis of the intensity of the labelling with pimonidazole and HIF1α was carried out by tracing around the spinal cord sections (white and grey matter, and grey matter alone, irrespectively), and measuring the pixel intensity using Image J (National Institute of Health, USA). Quantification of all other markers was carried out using the 'analyse particles' tool, on threshold images, to determine the number of positively labelled cells. The same settings were used for each image, and area measurements were carried out concurrently.

Grey matter atrophy in 2D: Transverse tissue sections stained with haematoxylin and eosin were microscopically examined (Zeiss Axiophot) and photographed (Nikon D300 camera). Grey matter atrophy was calculated by tracing around the area of the ventral horn grey matter on the lesion side and comparing it with the corresponding area on the contralateral side, expressing the result as a percentage relative to the non-lesioned contralateral grey matter. Quantification was carried out using the "analyse measurement" tool of Image J (National Institute of Health, USA).

Grey matter atrophy in 3D (MRI): Fixed spinal cords were washed in phosphate buffered saline prior to arrangement in a series of drinking straws positioned in a custom-built sample holder. The holder was positioned in a 20 cm horizontal 9.4T preclinical MRI scanner (Bruker Biospec, Billerica, MA) equipped with a 40 mm-diameter radiofrequency birdcage volume coil (Bruker Biospec, Billerica, MA). Images were acquired using a 3D fast low angle shot (FLASH) imaging sequence, with a spatial resolution of 30µm x 30µm x 300µm, flip angle 52.0°, matrix size 750 x 750 x 133, repetition time 100ms, time to echo 12ms and number of averages = four. From the images obtained, the cross-sectional area of the grey matter lesion was measured using the selection tool on Image J on each MRI slice where the lesion was visibly present. Subsequently, volume measurements were calculated using the slice thickness and cross-sectional values, where the lesion was visibly present, comparing the lesioned and contralateral sides to deduce the 3D-atrophy volume. A one-way ANOVA test was performed on atrophy volume results to compare the significance between different treatment groups and controls. For treatment groups compared at a single time point, t-tests were performed to assess significance.

Neuronal counts: Sections previously stained with cresyl violet were analysed using ImageJ software (National Institutes of Health, USA). Images of the sections were imported into ImageJ, and relevant regions of interest were identified. Using the cell counter plugin, cells exhibiting morphological features characteristic of motor neuron (such as large soma size, prominent nucleoli, and polygonal shape) were manually identified, counted, and tracked across sections. Counts and measurements were recorded for subsequent statistical analysis. All data were tested for normality using the ShapiroWilk test. A two-way ANOVA test was performed on neuronal counts to assess the significance between different time points and between the two treatment groups, those being saline and LPS. P-values of 0.05 (*), 0.01 (**), 0.001 (***) and 0.0001(****), were considered as statistically significant. All statistical analyses were carried out using GraphPad Prism version 8.

Statistical analyses were performed with Excel 2010 (Microsoft, USA), Prism 9.0 (GraphPad, USA) or Matlab, using Student's t-test, ANOVA or other related tests. The threshold for statistical significance was indicated as ns ($p>0.05$), * ($p\leq0.05$), ** ($p\leq0.01$), *** ($p\leq0.001$). All descriptive numbers in the text are shown as mean ± standard deviation (SD).

Mitochondrial biogenesis

Mitochondrial biogenesis was assessed as previously described[6]. Bromodeoxyuridine (BrdU) (Sigma-Aldrich, USA) in saline was maintained at 55°C for 30 minutes before intra-peritoneal injection (200mg/ml). The dosing volume was kept constant at 5ml/kg. Control animals were injected with saline. At termination, animals were perfused as described below, except that a piece of small intestine was harvested as a positive control for the BrdU (the inner crypt layer of the intestine undergoes rapid cell proliferation and is a good marker for DNA replication). BrdU was detected using fluorescence immunohistochemistry. Sections were re-fixed with a few drops of 4% PFA for 15 minutes at room temperature before PBS containing 0.3% TritonX-100 for five minutes at ambient temperature, and then the 2N hydrochloric acid for 90 minutes at 40°C. After washing with PBS, and then PBS containing 0.3% hydrogen peroxide for 10 minutes, followed by two washes with PBS for 5 minutes and then 5% goat serum in PBS with 0.1% Tween-20 for one hour. For mouse studies, rat anti-BrdU (MCA2060, AbD Serotec) was used at optimum concentration of 1:200,

along with rabbit anti VDAC1 (AbCam) at a concentration of 1:600. Slides were incubated with antibody solutions overnight at 4°C. The next morning, slides were washed stringently in troughs of PBS with 0.2% Tween 3 times, 15 minutes each at room temperature. Secondary antibodies in PBS were applied, biotinylated goat anti rat antibody at 1:200 (Vector labs) and goat anti rabbit conjugated to Alexa fluor 546 (Invitrogen) for 2 hours at room temperature. Slides were washed once again 3 times, each wash lasting 15 minutes with PBS and 0.2% tween. Finally, slides were incubated for 90 min at room temperature with Alexa conjugated Streptavidin (Invitrogen). After a final set of PBS washes, the slides were mounted using Vectashield (Vector labs) and then stored at 4°C until ready to be imaged.

Images were recorded with Pascal software (Zeiss) and analysed with Image J software (NCBI, USA). Each cell of interest was isolated and analysed individually for BrdU content in BrdU positive mitochondria. A threshold for fluorescence intensity was set manually and kept the same throughout the analysis. A 3D Object counter plug in was used to count the number of mitochondria that were labelled with BrdU. For each cell of interest, a Z project plug in was applied, and the area of BrdU positive particles was calculated per cell.

Tiled images from nerve sections were analysed for BrdU and porin co-localisation using a Coloc2 plug in. The image was split into two colour channels (porin in red and BrdU in green). The threshold was set manually in the red channel, keeping it consistent across all images. Co-localisation analysis was done in 3-dimensions. The level of co-localisation between BrdU-positive and porin-positive particles was presented as an average slope gradient.

Changes in pattern of mitochondrial biogenesis in short-time series experiments were analysed using GraphPad Prism6 software, a one-way ANOVA analysis ($p<0.05$).

Images were recorded with Pascal software (Zeiss) and analysed with Image J software (NCBI, USA). Each cell of interest was isolated and analysed individually for BrdU content in BrdU positive mitochondria. A threshold for fluorescence intensity was set manually and kept the same throughout the analysis. A 3D Object counter plug in was used to count the number of mitochondria that were labelled with BrdU. For each cell of interest, a Z project plug in was applied, and the area of BrdU positive particles was calculated per cell.

Tiled images from nerve sections were analysed for BrdU and porin co-localisation using a Coloc2 plug in. The image was split into two colour channels (porin in red and BrdU in green). The threshold was set manually in the red channel. Co-localisation analysis was done in 3-dimensions. The level of co-localisation between BrdU-positive and porin-positive particles was presented as an average slope gradient. Changes in pattern of mitochondrial biogenesis in short-time series experiments were analysed using GraphPad Prism6 software, a one-way ANOVA analysis ($p<0.5$).

Methods to detect and quantify mitochondrial complex IV [cytochrome c oxidase (COX)] and its catalytic component (COX-I) immunoreactivity. Combining complex IV histochemistry with immunohistochemistry

The complex IV histochemical reaction was carried out as stated previously[7]. After washing with PBS, immunohistochemistry for COX-1 was conducted by fixation in 4% paraformaldehyde, washing, and applying monoclonal antibodies raised against COX-I (IgG2a; Molecular Probes® Invitrogen Ltd., Paisley, UK). The Menapath X-Cell Plus HRP Polymer detection system (A. Menarini Diagnostics, Wokingham, UK) was used without a blocking step and an optimum concentration of 1:3200 for COX-I primary antibodies. Labelling was detected using the polymer kit as per manufacturing guidelines (A. Menarini Diagnostics, Wokingham, UK). The Vector® SG was used as the HRP substrate and the sections were dehydrated in graded ethanols. Histoclear was applied before mounting in DPX®.

Multi-spectral imaging and densitometric analysis of multiple chromogens:

Bright field images of the two chromogens (diaminobenzidine tetrahydrochloride and Vector® SG) were obtained using the Nuance imaging system (CRi, Woburn, MA), which uses liquid crystal tuneable filters to acquire multi-spectral images and deconvolution methods to separate or 'unmix' the original double labelled image to reveal unmixed images (in grey scale) of each chromogen[8]. The unmixed images may then be used to quantify each chromogen.

The global densitometric values of complex IV activity and immunoreactivity in the entire ventral horns were determined by manually outlining the ventral horns in unmixed images. The single cells with abundant mitochondria, which are mostly neurons based on morphology and NeuN labelling (not shown), were outlined in ventral horns of rat spinal cords by setting a fixed threshold on COX-I immunoreactivity in the sections subjected to immunohistochemistry only. The threshold of COX-I immunoreactivity, which was kept constant, was used to outline the cells abundant in mitochondria in the unmixed images of sections subjected to both complex IV histochemistry and immunohistochemistry. The mean signal and the area of immunoreactivity were recorded within each cell. The outline of cells, based on the immunoreactivity above the threshold, was copied on to the unmixed images of complex IV activity and the mean signal intensity of complex IV histochemistry (activity) was recorded within each corresponding cell.

Discussion

Relevance to neurological disease

We do not advance the new lesion as a precise model of any particular neurological disease, but rather as a tractable model that exhibits mechanisms likely to contribute to the slowly progressive degeneration and atrophy observed in

neurological diseases. However, there are notable similarities between the model and progressive MS. In both the model and secondary progressive MS there is i) innate hypoxic neuroinflammation (provoked by LPS in the model, and potentially by acquired immune activity in MS) accompanied by acute disability (relapse) in young adulthood, which undergoes remission, but is ii) followed over the adult lifetime by progressive disability, neurodegeneration and atrophy, which iii) develop in conjunction with astroglial and microglial activation and a deficiency of mitochondrial respiratory complex IV.

The key role of hypoxia is interesting when considered in conjunction with previous observations, because inflammatory tissue hypoxia is emerging as a key cause of both acute and progressive disability and smouldering neurodegeneration and atrophy[this study and 5,9], as well as demyelination[10], all cardinal features of MS. In experimental autoimmune encephalomyelitis (EAE), a commonly used model of MS, hypoxia in the acutely inflamed lesions is sufficient to cause the impairment in mitochondrial function[11], resulting in neuronal inexcitability and disability. Thus, animals dragging their hindlimbs at the onset of inflammatory disease expression in EAE can be restored to strong walking within two hours of breathing normobaric raised oxygen, and the neurological deficits return within one hour of breathing room air[5,9,12].

These dramatic and swift effects in EAE are strongly supportive of a direct effect of tissue hypoxia in impairing mitochondrial function. Impairment by lack of oxygen is probably an important factor in the acute phase of the new model lesion, but the reduction in COX:COX1 activity suggests additional direct damage to mitochondrial complex IV, as may result from the combined effects of hypoxia, nitric oxide and superoxide[12], and the consequent formation of the potent oxidising agent peroxynitrite. Peroxynitrite can cause direct nitration and damage of mitochondrial complex IV[13] and also damage the mitochondrial or nuclear DNA responsible for complex IV formation[14]. The acute neurological deficits probably arise from hypoperfusion of the inflamed tissue, perhaps due in part to raised levels of the potent vasoconstrictive agent endothelin-1, as occurs in MS[15]: nimodipine is a very effective vasodilating antagonist of endothelin-1. Aside from causing neurological deficits, inflammatory tissue hypoxia also causes the Pattern III type of demyelination[12] that occurs in early MS lesions[16], and again strategies that maintain tissue oxygenation provide effective treatments[12]. We conclude that prompt therapy to avoid tissue hypoxia, such as when it is signalled by the onset of new relapses, may achieve significant benefits in MS by reducing both acute and progressive disability.

Aside from MS[17], features including innate immune activation and hypoperfusion precede long term cognitive and other deficits in, e.g. sport-related concussion[18] and boxing[19], traumatic brain injury[20], and Alzheimer's disease[21]. The current findings suggest that precautions to maintain cerebral perfusion and oxygenation, such as those employed in this study, may be important in providing protection from later disability.

Supplementary Table 1- Immunohistochemistry

Antibody	Target	Isotype	Blocking Buffer	Dilution	Source
**Mouse Anti-GFAP**	Astrocytes	Rabbit IgG	5% normal horse serum (Sigma) in 0.01% PBS-triton-x	1:200	Sigma
**Mouse Hydroxyprobe-1-anti-pimonidazole**	Pimonidazole adducts	Mouse IgG	0.25% casein (VWR International, UK), in 0.01% PBS- triton-x	1:500	HPI Inc
**Mouse Anti-ED1**	Activated macrophages/ microglia	Mouse IgG1	5% normal horse serum (Sigma) in 0.01% PBS-triton-x	1:200	Abcam
**Rabbit anti-IBA**	Macrophages/ Microglia	Rabbit IgG	5% normal goat serum (Sigma) in 0.01% PBS-triton-x	1:500	WAKO
**Rabbit Anti-HIF-1α**	Hypoxia inducible factor-1 α	Rabbit IgG	5% normal goat serum (Sigma) in 0.01% PBS-triton-x	1:200	Abcam

Supplementary Table 2- Immunofluorescence

Antibody	Target	Isotype	Blocking Buffer	Dilution	Source
**Rabbit Anti- GFAP**	Astrocytes	Rabbit IgG	5% NGS in 0.01% PBS-triton	1:500	DAKO
**Rabbit Hydroxyprobe-1-anti-pimonidazole**	Pimonidazole adducts	Rabbit IgG	5% NGS in 0.01% PBS-triton	1:500	HPI Inc
**Rabbit Anti-IBA**	Microglia/ Macrophages	Rabbit IgG	5% NGS in 0.01% PBS-triton	1:500	Abcam
**Rabbit anti-Fibrinogen**	Fibrinogen	Rabbit IgG	5% NGS in 0.01% PBS-triton	1:400	Abcam
**Rabbit Anti-iNOS**	iNOS	Rabbit IgG	5% NGS in 0.01% PBS-triton	1:100	Invitrogen

1. Sandiego CM, Gallezot JD, Pittman B*, et al*. Imaging robust microglial activation after lipopolysaccharide administration in humans with PET. *Proc Natl Acad Sci U S A*. Oct 6 2015;112(40):12468-73. doi:10.1073/pnas.1511003112
2. Buscarinu MC, Cerasoli B, Annibali V*, et al*. Altered intestinal permeability in patients with relapsing-remitting multiple sclerosis: A pilot study. *Mult Scler*. Mar 2017;23(3):442-446. doi:10.1177/1352458516652498
3. Lakes EH, Allen KD. Gait analysis methods for rodent models of arthritic disorders: reviews and recommendations. *Osteoarthritis Cartilage*. Nov 2016;24(11):1837-1849. doi:10.1016/j.joca.2016.03.008
4. MacKenzie SJ, Yi JL, Singla A, Russell TM, Calancie B. Innervation and function of rat tail muscles for modeling cauda equina injury and repair. *Muscle Nerve*. Jul 2015;52(1):94-102. doi:10.1002/mus.24498
5. Davies AL, Desai RA, Bloomfield PS*, et al*. Neurological deficits caused by tissue hypoxia in neuroinflammatory disease. *Ann Neurol*. 12/2013 2013;74(6):815-825. doi:10.1002/ana.24006 [doi]
6. Desai R. A study of mitochondrial biogenesis in the rodent nervous system. Ph.D. Thesis. University College London; 2015.
7. Campbell GR, Mahad DJ. A Method to Detect Cytochrome c Oxidase Activity and Mitochondrial Proteins in Oligodendrocytes. *Methods Mol Biol*. 2019;1936:333-342. doi:10.1007/978-1-4939-9072-6_19
8. Levenson RM, Mansfield JR. Multispectral imaging in biology and medicine: slices of life. *Cytometry A*. Aug 1 2006;69(8):748-58. doi:10.1002/cyto.a.20319
9. Amatruda M, Harris K, Matis A*, et al*. Oxygen treatment reduces neurological deficits and demyelination in two animal models of multiple sclerosis. *Neuropathol Appl Neurobiol*. Dec 15 2023;49:e12868. doi:10.1111/nan.12868
10. Desai RA, Davies AL, Tachrount M*, et al*. Cause and prevention of demyelination in a model multiple sclerosis lesion. *Ann Neurol*. 1/27/2016 2016;79:591-604. doi:10.1002/ana.24607 [doi]
11. Sadeghian M, Mastrolia V, Haddad AR*, et al*. Mitochondrial dysfunction is an important cause of neurological deficits in an inflammatory model of multiple sclerosis. *Scientific Reports*. 2016 2016;6:33249.
12. Desai RA, Davies AL, Del Rossi N*, et al*. Nimodipine reduces dysfunction and demyelination in models of multiple sclerosis. *Ann Neurol*. Jul 2020;88(1):123-136. doi:10.1002/ana.25749
13. Parihar A, Vaccaro P, Ghafourifar P. Nitric oxide irreversibly inhibits cytochrome oxidase at low oxygen concentrations: evidence for inverse oxygen concentration-dependent peroxynitrite formation. *Iubmb Life*. 1/2008 2008;60(1):64-67. doi:10.1002/iub.12 [doi]
14. Calcerrada P, Peluffo G, Radi R. Nitric oxide-derived oxidants with a focus on peroxynitrite: molecular targets, cellular responses and therapeutic implications. *Curr Pharm Des*. Dec 2011;17(35):3905-32. doi:10.2174/138161211798357719
15. Monti L, Morbidelli L, Bazzani L, Rossi A. Influence of Circulating Endothelin-1 and Asymmetric Dimethylarginine on Whole Brain Circulation Time in Multiple Sclerosis. *Biomark Insights*. 2017;12:1177271917712514. doi:10.1177/1177271917712514
16. Aboul-Enein F, Lassmann H. Mitochondrial damage and histotoxic hypoxia: a pathway of tissue injury in inflammatory brain disease? *Acta Neuropathol (Berl)*. 1/2005 2005;109(1):49-55.
17. Mascali D, Villani A, Chiarelli AM*, et al*. Pathophysiology of multiple sclerosis damage and repair: Linking cerebral hypoperfusion to the development of irreversible tissue loss in multiple sclerosis using magnetic resonance imaging. *Eur J Neurol*. Aug 2023;30(8):2348-2356. doi:10.1111/ene.15827
18. Owens TS, Calverley TA, Stacey BS*, et al*. Contact events in rugby union and the link to reduced cognition: evidence for impaired redox-regulation of cerebrovascular function. *Exp Physiol*. Sep 2021;106(9):1971-1980. doi:10.1113/EP089330
19. Bailey DM, Jones DW, Sinnott A*, et al*. Impaired cerebral haemodynamic function associated with chronic traumatic brain injury in professional boxers. *Clin Sci (Lond)*. Feb 2013;124(3):177-89. doi:10.1042/CS20120259
20. Salehi A, Zhang JH, Obenaus A. Response of the cerebral vasculature following traumatic brain injury. *J Cereb Blood Flow Metab*. Jul 2017;37(7):2320-2339. doi:10.1177/0271678X17701460
21. Sweeney MD, Kisler K, Montagne A, Toga AW, Zlokovic BV. The role of brain vasculature in neurodegenerative disorders. *Nat Neurosci*. Oct 2018;21(10):1318-1331. doi:10.1038/s41593-018-0234-x
