# Supplementary material for: A cause and protective treatment for acute and progressive disability and grey matter atrophy
Source: Brain. 2025 Dec 15;149(4):1144–52. doi: 10.1093/brain/awaf465 (PMC13058463; doi:10.1093/brain/awaf465)
Supplement: awaf465_Supplementary_Data [file awaf465_supplementary_data.zip › brain-2025-00625-File005.pdf]

Supplementary Figure 1

A. Graph showing the progression of disability over a year for animals intraspinally injected with LPS and tested while walking on a horizontal surface for 1 metre, during acute and chronic periods. Animals receiving no treatment (n=11) show significantly greater disability than animals acutely treated (for 4 days) with either inspiratory 80% oxygen (n=10) or nimodipine (n=18). Animals were tested before injection, for four consecutive days after injection, and then monthly. Data show a separate trial from that illustrated in Fig. 3A. Mean ± SEM; one-way ANOVA, Dunn's multiple comparisons test, hyperoxia $p=0.0031$, nimodipine $p<0.0001$.

B. Graph showing the area occupied by activated $ED1^{+}$ microglia/macrophages two days after intraspinal LPS injection vs. saline control injection, comparing the ipsilateral and contralateral ventral grey matter. The ipsilateral ventral grey matter injected with LPS contained significantly more activated microglia/macrophages than either the contralateral grey matter or the grey matter of saline injected controls. Ipsilateral saline vs LPS = independent t test, $p<0.001$; LPS ipsilateral vs contralateral = paired t test $p<0.001$.

C. Graph showing the relative intensity of tissue hypoxia marked by immunolabelling for pimonidazole, comparing the ventral grey matter of the spinal cord ipsilateral to an injection of either LPS or saline (control) one to four days previously. The LPS-injected spinal cord labels significantly more intensely for hypoxia than control spinal cord two days following injection. Independent t test, $p<0.001$.

D. Graph showing the relative intensity of tissue hypoxia marked by immunolabelling for hypoxia inducible factor-1a (HIF1a), comparing the ventral grey matter of the spinal cord ipsilateral to an injection of either LPS or saline (control) two days previously. The LPS-injected spinal cord labels significantly more intensely for hypoxia than control spinal cord. Independent t test, $p<0.05$.

E. Graph showing the area occupied by immunolabelling for the inducible form of nitric oxide synthase (iNOS), comparing the spinal ventral grey matter ipsilateral and contralateral to an injection of either LPS or saline (control) two days previously. The spinal cord ipsilateral to the LPS injection labels significantly more intensely for iNOS than the contralateral side of the same animals ($p<0.01$), and significantly more intensely than saline-injected controls ($p<0.05$). Ipsilateral saline vs LPS = independent t test; LPS ipsilateral vs contralateral = paired t test.

F. Graph showing breakdown of the BBB marked by immunolabelling for fibrinogen, comparing the spinal grey matter ipsilateral and contralateral to an injection of either LPS or saline (control) two days previously. The spinal cord ipsilateral to the LPS injection labels significantly more intensely for fibrinogen than the contralateral side of the same animals ($p<0.001$), and significantly more intensely than saline-injected controls ($p<0.05$). Ipsilateral saline vs LPS = independent t test; LPS ipsilateral vs contralateral = paired t test.

G. Images showing COX activity and COX-I immunoreactivity in the ventral horn of a control animal (Gi) and the ventral horn of an animal injected with LPS (Gii). Red labelling indicates complex IV activity in the ventral horn, judged by COX histochemistry. Green labelling indicates immunoreactivity of the complex IV subunit-I (COX-I) in mitochondria, in particular mitochondria that lack complex IV activity, following sequential COX histochemistry/COX-I immunohistochemistry as previously described (Mahad et al., J. Neurosci. Methods 2009;184:310-319). Ventral horn neurons abundant in complex IV activity are present in control tissue (red and minimal green labelling) whereas complex IV is deficient in the LPS-injected ventral horn (minimal red and mostly green labelling). Arrowheads indicate ventral horn neuronal cell bodies.

H. Bar graph showing lack of effect of acute treatment with 80% oxygen or nimodipine in preventing acute inflammation, as indicated by immunolabeling for IBA1. Mean ± SD; one-way ANOVA, no statistical significance.

I. Gait analysis showing the balance between the hindlimbs two days after the intraspinal injection of LPS. In placebo-treated animals the function of the hindlimb ipsilateral to the lesion was significantly worse than that of the contralateral limb (i.e. gait balance significantly below zero), but the function of the ipsilateral leg was protected in animals acutely treated with either 80% oxygen or nimodipine for 4 days (i.e. function in the ipsilateral hindlimb was not significantly different from that of the contralateral hindlimb). Mean ± SEM; one-way ANOVA, Dunn's multiple comparisons test, no statistical significance.

Ji. The length of the lesion assessed by MRI examination one year after induction, comparing lesions in the absence of neuroprotective treatment (n=14), and after acute (four days) treatment with inspiratory oxygen (80%) (n=7) or nimodipine (n=16). Lesions are significantly shorter in treated animals (hyperoxia $p=0.0037$; nimodipine $p=0.0235$). Mean ±SD; two-way ANOVA, Dunnett's multiple comparisons test.

Jii. Gait analysis showing the swing speed scores for the hindlimbs ipsilateral and contralateral to an LPS lesion induced 6 months previously. Lines connect the legs of individual animals, grouped into no treatment, and acute treatment with nimodipine or 80% oxygen for the first 4

dpi. The swing speed was significantly slower on the ipsilateral side with no treatment, but either treatment provided protection (i.e. no significant difference between sides). Paired t-test; $p=0.0270$.

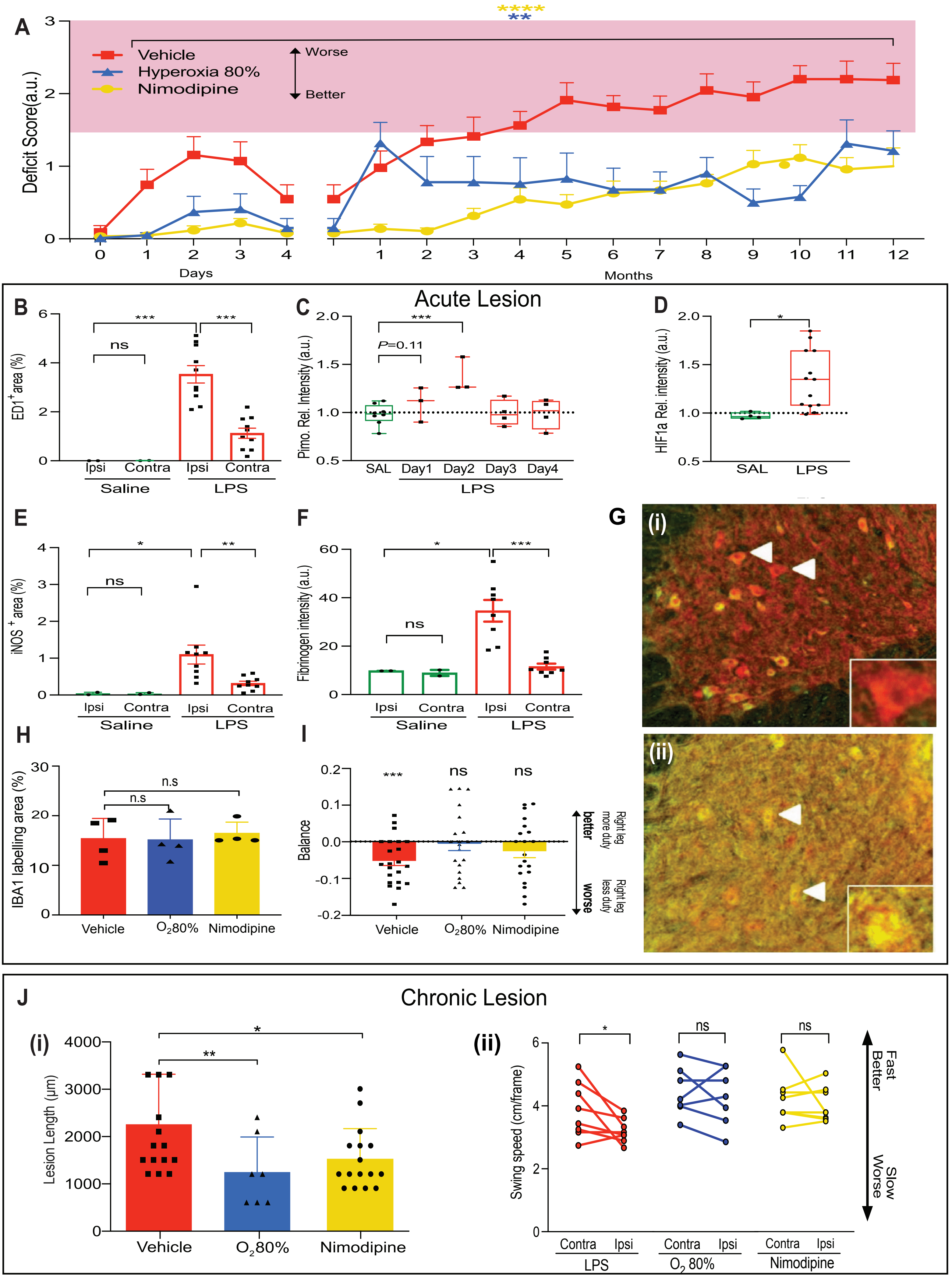

A
Vehicle
Hyperoxia 80%
Nimodipine
Worse
Better
Deficit Score(a.u.)
Days
Months
B
ED1+area (%)
Saline
LPS
Ipsi
Contra
C
Acute Lesion
Pimo. Rel. Intensity (a.u.)
P=0.11
SAL
Day1
Day2
Day3
Day4
D
HIF1a Rel. intensity (a.u.)
E
iNOS + area (%)
F
Fibrinogen intensity (a.u.)
G
(i)
(ii)
H
IBA1 labelling area (%)
Vehicle
O280%
Nimodipine
I
Balance
better
worse
Right leg more duty
Right leg less duty
J
Chronic Lesion
(i)
Lesion Length (μm)
(ii)
Swing speed (cm/frame)
Fast Better
Slow Worse
O2 80%
